# (4-Amino­benzene­sulfonato)­hepta­aqua­gadolinium(III) 4-amino­benzene­sulfonate nitrate 4,4′-bipyridyl tetra­solvate dihydrate

**DOI:** 10.1107/S1600536810020520

**Published:** 2010-06-05

**Authors:** Lujiang Hao, Xiaofei Zhang, Jiangkui Chen

**Affiliations:** a Shandong Provincial Key Laboratory of Microbial Engineering, Shandong Institute of Light Industry, Jinan 250353, People’s Republic of China

## Abstract

In the title compound, [Gd(C_6_H_6_O_3_S)(H_2_O)_7_](C_6_H_6_O_3_S)(NO_3_)·4C_10_H_8_N_2_·2H_2_O, the Gd^III^ ion is octa­coordinated by seven water mol­ecules and one O-bonded 4-amino­benzene­sulfonate anion in a square-anti­prismatic arrangement. In the crystal, the components are linked by N—H⋯O, O—H⋯N and O—H⋯O hydrogen bonds.

## Related literature

For background to lanthanide coordination networks, see: Karthikeyan *et al.* (1989 ▶).
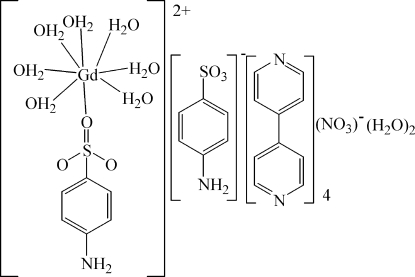


## Experimental

### 

#### Crystal data

[Gd(C_6_H_6_O_3_S)(H_2_O)_7_](C_6_H_6_O_3_S)(NO_3_)·4C_10_H_8_N_2_·2H_2_O
*M* *_r_* = 1350.50
Orthorhombic, 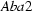

*a* = 33.529 (2) Å
*b* = 23.3375 (10) Å
*c* = 15.2046 (10) Å
*V* = 11897.3 (12) Å^3^
*Z* = 8
Mo *K*α radiation
μ = 1.26 mm^−1^
*T* = 296 K
0.12 × 0.10 × 0.08 mm

#### Data collection

Bruker APEXII CCD diffractometer
Absorption correction: multi-scan (*SADABS*; Bruker, 2001 ▶) *T*_min_ = 0.863, *T*_max_ = 0.906
41191 measured reflections
10477 independent reflections
8881 reflections with *I* > 2σ(*I*)
*R*_int_ = 0.045

#### Refinement

*R*[*F* ^2^ > 2σ(*F* ^2^)] = 0.036
*wR*(*F* ^2^) = 0.088
*S* = 1.00
10477 reflections
811 parameters
27 restraints
H atoms treated by a mixture of independent and constrained refinement
Δρ_max_ = 0.35 e Å^−3^
Δρ_min_ = −0.53 e Å^−3^
Absolute structure: Flack (1983 ▶), 5009 Friedel pairs
Flack parameter: 0.002 (1)

### 

Data collection: *APEX2* (Bruker, 2004 ▶); cell refinement: *SAINT-Plus* (Bruker, 2001 ▶); data reduction: *SAINT-Plus*; program(s) used to solve structure: *SHELXS97* (Sheldrick, 2008 ▶); program(s) used to refine structure: *SHELXL97* (Sheldrick, 2008 ▶); molecular graphics: *SHELXTL* (Sheldrick, 2008 ▶); software used to prepare material for publication: *SHELXTL*.

## Supplementary Material

Crystal structure: contains datablocks global, I. DOI: 10.1107/S1600536810020520/hb5454sup1.cif

Structure factors: contains datablocks I. DOI: 10.1107/S1600536810020520/hb5454Isup2.hkl

Additional supplementary materials:  crystallographic information; 3D view; checkCIF report

## Figures and Tables

**Table 1:** Selected bond lengths (Å)

Gd1—O6*W*	2.375 (4)
Gd1—O2*W*	2.373 (4)
Gd1—O1*W*	2.389 (4)
Gd1—O3*W*	2.392 (4)
Gd1—O7*W*	2.391 (4)
Gd1—O5*W*	2.401 (4)
Gd1—O1	2.434 (4)
Gd1—O4*W*	2.440 (4)

**Table 2:** Hydrogen-bond geometry (Å, °)

*D*—H⋯*A*	*D*—H	H⋯*A*	*D*⋯*A*	*D*—H⋯*A*
O1*W*—H1*W*⋯N6	0.82 (2)	2.12 (2)	2.770 (7)	136 (3)
O1*W*—H2*W*⋯O5	0.82 (2)	2.13 (1)	2.759 (6)	134 (3)
O2*W*—H3*W*⋯O8*W*	0.82 (2)	1.93 (1)	2.657 (7)	147 (2)
O3*W*—H6*W*⋯N8	0.82 (2)	1.99 (1)	2.728 (7)	149 (2)
O4*W*—H8*W*⋯N9^i^	0.82 (2)	2.19 (2)	2.807 (7)	133 (1)
O5*W*—H9*W*⋯N4^ii^	0.82 (1)	1.86 (1)	2.647 (7)	159 (2)
O5*W*—H10*W*⋯O3	0.82 (2)	2.51 (2)	3.236 (6)	148 (4)
O5*W*—H10*W*⋯O1	0.82 (2)	2.50 (3)	2.931 (5)	114 (2)
O6*W*—H11*W*⋯O3^iii^	0.82 (3)	1.95 (3)	2.765 (6)	175 (5)
O6*W*—H12*W*⋯N3	0.82 (1)	1.90 (1)	2.719 (7)	178 (8)
O7*W*—H13*W*⋯N1^iv^	0.82 (3)	2.19 (2)	2.902 (7)	145 (3)
O7*W*—H14*W*⋯N5^ii^	0.82 (1)	2.37 (4)	2.758 (7)	110 (3)
O7*W*—H14*W*⋯O3*W*	0.82 (1)	2.29 (1)	2.709 (6)	112 (3)
O8*W*—H16*W*⋯N11^v^	0.82 (3)	1.98 (3)	2.798 (9)	176 (6)
O9*W*—H17*W*⋯O20	0.82 (3)	2.06 (4)	2.873 (7)	169 (6)
O9*W*—H18*W*⋯O2^vi^	0.82 (4)	2.24 (5)	3.028 (7)	161 (7)
N1—H1*A*⋯O6^vi^	0.86	2.22	2.972 (7)	146
N1—H1*B*⋯O2^vi^	0.86	2.14	2.958 (6)	159
N7—H7*B*⋯O14^vii^	0.86	2.51	3.289 (12)	151
N7—H7*A*⋯O15^viii^	0.86	2.63	3.345 (12)	141
N7—H7*A*⋯O16^viii^	0.86	2.46	3.302 (13)	167

Symmetry codes: (i) 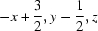; (ii) 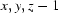; (iii) 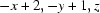; (iv) 
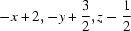
; (v) 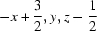; (vi) 
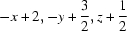
; (vii) 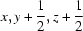; (viii) 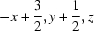.

**Fig. 1.**
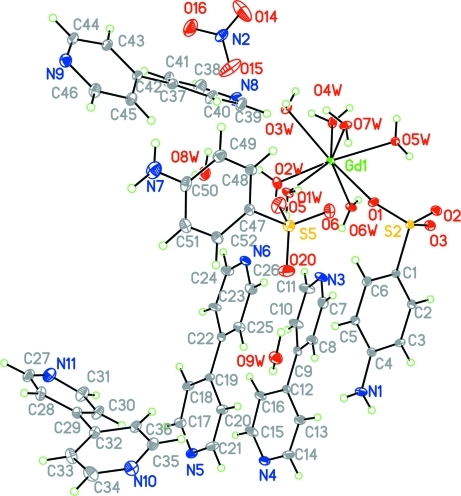
The building blocks of (I) with displacement ellipsoids drawn at the 30% probability level; H atoms are given as spheres of arbitrary radius.

**Fig. 2.**
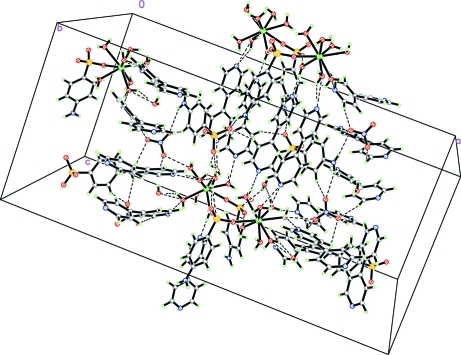
The crystal packing of (I), displayed with N—H···O and O—H···O hydrogen bonds as dashed lines.

## supplementary crystallographic information

### Comment

The design and synthesis of metal-organic compounds has attracted continuous research interest not only because of their appealing structural and topological novelties, but also due to their interesting optical, electronic, magnetic, and catalytic properties, as well as their potential medical applications (Karthikeyan *et al.*, 1989). Here, we describe the synthesis and structural characterization of the title compound.

As shown in Figure 1, Gd(III) is octacoordinated by senven water molecules and one *p*-amino-benzenesulfonate anion. The Gd—O bond lengths are in the range of 2.370 (4)—2.439 (4) Å. In the molecule, one *p*-amino-benzenesulfonate, one nitrate, and two water molecules disassociate. N—H···O2, N—H···S, O—H···N, O—H···N, O—H···O hydrogen bonding between the cationic and anionic moieties and the uncoordinated water molecules leads to a consolidation of the structure (Fig. 2; Table 2).

### Experimental

A mixture of 4-aminobenzene sulfonic acid (1 mmol 0.17 g), gadolinium(III) nitrate hexahydrate (0.5 mmol, 0.17 g), and 4,4-bipyridine (1 mmol, 0.14 g) in 10 ml distilled water was sealed in a 25 ml Teflon-lined stainless steel autoclave and was kept at 433 K for three days. Colourless blocks of (I) were obtained upon cooling. Anal. C_52_H_62_GdN_11_O_18_S_2_: C, 46.22; H, 4.59; N, 11.41. Found: C, 46.01; H, 4.48; N, 11.23%.

### Refinement

All hydrogen atoms bound to aromatic carbon atoms were refined in calculated positions using a riding model with a C—H distance of 0.93 Å and *U*_iso_ = 1.2*U*_eq_(C). The hydrogen atoms bound to N atoms were refined in calculated positions using a riding model with a N—H distance of 0.86 Å and *U*_iso_ = 1.2*U*_eq_(C). Water molecules are refined by using the '*DFIX*' command with the hydrogen atoms were separated with 1.38 Å, and the lengths of bond H—O were constrained with 0.82 Å with error 0.02 and *U*_iso_ = 1.5*U*_eq_ (O).   The location of the water H atoms should be regarded as less certain that those of the other H atoms.

### Crystal data

**Table:**

[Gd(C_6_H_6_O_3_S)(H_2_O)_7_](C_6_H_6_O_3_S)(NO_3_)·4C_10_H_8_N_2_·2H_2_O	*F*(000) = 5528
*M**_r_* = 1350.50	*D*_x_ = 1.508 Mg m^−^^3^
Orthorhombic, *A**b**a*2	Mo *K*α radiation, λ = 0.71073 Å
Hall symbol: A 2 -2ac	Cell parameters from 10477 reflections
*a* = 33.529 (2) Å	θ = 2.4–25.0°
*b* = 23.3375 (10) Å	µ = 1.26 mm^−^^1^
*c* = 15.2046 (10) Å	*T* = 296 K
*V* = 11897.3 (12) Å^3^	Block, colourless
*Z* = 8	0.12 × 0.10 × 0.08 mm

### Data collection

**Table:**

Bruker APEXII CCD diffractometer	10477 independent reflections
Radiation source: fine-focus sealed tube	8881 reflections with *I* > 2σ(*I*)
graphite	*R*_int_ = 0.045
phi and ω scans	θ_max_ = 25.0°, θ_min_ = 2.4°
Absorption correction: multi-scan (*SADABS*; Bruker, 2001)	*h* = −39→39
*T*_min_ = 0.863, *T*_max_ = 0.906	*k* = −27→25
41191 measured reflections	*l* = −18→18

### Refinement

**Table:**

Refinement on *F*^2^	Secondary atom site location: difference Fourier map
Least-squares matrix: full	Hydrogen site location: inferred from neighbouring sites
*R*[*F*^2^ > 2σ(*F*^2^)] = 0.036	H atoms treated by a mixture of independent and constrained refinement
*wR*(*F*^2^) = 0.088	*w* = 1/[σ^2^(*F*_o_^2^) + (0.044*P*)^2^ + 12.9607*P*] where *P* = (*F*_o_^2^ + 2*F*_c_^2^)/3
*S* = 1.00	(Δ/σ)_max_ = 0.001
10477 reflections	Δρ_max_ = 0.35 e Å^−^^3^
811 parameters	Δρ_min_ = −0.53 e Å^−^^3^
27 restraints	Absolute structure: Flack (1983), 5009 Friedel pairs
Primary atom site location: structure-invariant direct methods	Flack parameter: 0.002 (1)

### Special details

**Table:**

Geometry. All e.s.d.'s (except the e.s.d. in the dihedral angle between two l.s. planes) are estimated using the full covariance matrix. The cell e.s.d.'s are taken into account individually in the estimation of e.s.d.'s in distances, angles and torsion angles; correlations between e.s.d.'s in cell parameters are only used when they are defined by crystal symmetry. An approximate (isotropic) treatment of cell e.s.d.'s is used for estimating e.s.d.'s involving l.s. planes.
Refinement. Refinement of *F*^2^ against ALL reflections. The weighted *R*-factor *wR* and goodness of fit *S* are based on *F*^2^, conventional *R*-factors *R* are based on *F*, with *F* set to zero for negative *F*^2^. The threshold expression of *F*^2^ > σ(*F*^2^) is used only for calculating *R*-factors(gt) *etc*. and is not relevant to the choice of reflections for refinement. *R*-factors based on *F*^2^ are statistically about twice as large as those based on *F*, and *R*- factors based on ALL data will be even larger.

### Fractional atomic coordinates and isotropic or equivalent isotropic displacement parameters (Å^2^)

**Table:**

	*x*	*y*	*z*	*U*_iso_*/*U*_eq_	
C1	1.01881 (15)	0.6735 (2)	0.4126 (3)	0.0317 (11)	
C2	1.04516 (16)	0.6502 (2)	0.4716 (3)	0.0373 (12)	
H2	1.0613	0.6198	0.4545	0.045*	
C3	1.04792 (18)	0.6714 (3)	0.5561 (4)	0.0423 (14)	
H3	1.0658	0.6549	0.5955	0.051*	
C4	1.02425 (16)	0.7174 (2)	0.5830 (3)	0.0366 (13)	
C5	0.99778 (17)	0.7407 (2)	0.5226 (3)	0.0391 (14)	
H5	0.9818	0.7714	0.5391	0.047*	
C6	0.99492 (16)	0.7190 (2)	0.4389 (4)	0.0392 (13)	
H6	0.9768	0.7350	0.3995	0.047*	
C7	0.9597 (2)	0.5837 (3)	0.5639 (5)	0.065 (2)	
H7	0.9668	0.6149	0.5294	0.078*	
C8	0.9629 (2)	0.5891 (3)	0.6540 (5)	0.058 (2)	
H8	0.9726	0.6230	0.6781	0.069*	
C9	0.9520 (2)	0.5445 (3)	0.7085 (4)	0.0402 (16)	
C10	0.9388 (3)	0.4959 (3)	0.6658 (4)	0.069 (2)	
H10	0.9316	0.4638	0.6983	0.083*	
C11	0.9361 (3)	0.4947 (4)	0.5753 (5)	0.083 (3)	
H11	0.9259	0.4618	0.5492	0.099*	
C12	0.95518 (19)	0.5476 (3)	0.8045 (4)	0.0364 (15)	
C13	0.9811 (2)	0.5832 (3)	0.8464 (5)	0.065 (2)	
H13	0.9976	0.6072	0.8139	0.077*	
C14	0.9829 (3)	0.5837 (4)	0.9365 (5)	0.077 (3)	
H14	1.0010	0.6084	0.9630	0.092*	
C15	0.9355 (3)	0.5184 (4)	0.9479 (5)	0.074 (3)	
H15	0.9195	0.4950	0.9826	0.089*	
C16	0.9307 (2)	0.5152 (3)	0.8572 (5)	0.063 (2)	
H16	0.9114	0.4915	0.8327	0.075*	
C17	0.8646 (2)	0.6318 (3)	0.9382 (5)	0.056 (2)	
H17	0.8504	0.6059	0.9726	0.067*	
C18	0.8591 (2)	0.6306 (3)	0.8485 (5)	0.053 (2)	
H18	0.8414	0.6042	0.8245	0.064*	
C19	0.87929 (19)	0.6675 (3)	0.7936 (4)	0.0357 (16)	
C20	0.9046 (2)	0.7060 (3)	0.8358 (5)	0.055 (2)	
H20	0.9189	0.7329	0.8036	0.066*	
C21	0.9079 (2)	0.7037 (4)	0.9264 (5)	0.063 (2)	
H21	0.9250	0.7300	0.9529	0.076*	
C22	0.87594 (19)	0.6666 (3)	0.6959 (5)	0.0395 (17)	
C23	0.8567 (2)	0.6225 (3)	0.6532 (4)	0.056 (2)	
H23	0.8445	0.5933	0.6850	0.067*	
C24	0.8556 (3)	0.6222 (4)	0.5622 (6)	0.068 (2)	
H24	0.8428	0.5918	0.5345	0.081*	
C25	0.8918 (2)	0.7093 (3)	0.6430 (5)	0.0504 (18)	
H25	0.9044	0.7407	0.6685	0.060*	
C26	0.8890 (2)	0.7054 (3)	0.5529 (5)	0.058 (2)	
H26	0.9000	0.7347	0.5191	0.070*	
C27	0.7098 (2)	0.7503 (4)	0.9084 (5)	0.077 (2)	
H27	0.6825	0.7551	0.9003	0.092*	
C28	0.7333 (2)	0.7988 (3)	0.9122 (5)	0.066 (2)	
H28	0.7220	0.8349	0.9062	0.079*	
C29	0.77372 (19)	0.7930 (3)	0.9250 (4)	0.0522 (16)	
C30	0.7882 (2)	0.7383 (3)	0.9354 (5)	0.069 (2)	
H30	0.8153	0.7322	0.9454	0.083*	
C31	0.7622 (2)	0.6925 (3)	0.9310 (6)	0.083 (3)	
H31	0.7725	0.6559	0.9396	0.099*	
C32	0.80115 (18)	0.8427 (3)	0.9256 (4)	0.0506 (15)	
C33	0.7922 (2)	0.8937 (3)	0.9665 (5)	0.072 (2)	
H33	0.7683	0.8983	0.9969	0.087*	
C34	0.8197 (3)	0.9385 (3)	0.9614 (6)	0.078 (2)	
H34	0.8135	0.9725	0.9903	0.094*	
C35	0.8615 (2)	0.8864 (3)	0.8783 (5)	0.0587 (18)	
H35	0.8851	0.8836	0.8464	0.070*	
C36	0.8371 (2)	0.8394 (3)	0.8811 (5)	0.0566 (18)	
H36	0.8446	0.8055	0.8534	0.068*	
C37	0.7417 (2)	0.7246 (3)	0.1777 (6)	0.071 (2)	
H37	0.7148	0.7151	0.1814	0.086*	
C38	0.7696 (2)	0.6819 (3)	0.1772 (6)	0.081 (2)	
H38	0.7609	0.6442	0.1805	0.097*	
C39	0.8195 (2)	0.7462 (4)	0.1683 (5)	0.072 (2)	
H39	0.8466	0.7542	0.1654	0.087*	
C40	0.79343 (19)	0.7909 (3)	0.1685 (5)	0.0617 (18)	
H40	0.8031	0.8282	0.1655	0.074*	
C41	0.75276 (18)	0.7816 (3)	0.1729 (4)	0.0501 (15)	
C42	0.72368 (16)	0.8286 (3)	0.1737 (4)	0.0444 (14)	
C43	0.68574 (18)	0.8222 (3)	0.1372 (4)	0.0564 (17)	
H43	0.6784	0.7881	0.1101	0.068*	
C44	0.6595 (2)	0.8671 (4)	0.1419 (5)	0.063 (2)	
H44	0.6344	0.8620	0.1170	0.075*	
C45	0.73194 (17)	0.8813 (3)	0.2124 (4)	0.0541 (17)	
H45	0.7568	0.8881	0.2376	0.065*	
C46	0.7029 (2)	0.9237 (3)	0.2131 (5)	0.063 (2)	
H46	0.7090	0.9587	0.2389	0.075*	
C47	0.87083 (17)	0.8856 (2)	0.3376 (4)	0.0432 (14)	
C48	0.86031 (16)	0.9022 (2)	0.2554 (6)	0.0486 (14)	
H48	0.8721	0.8845	0.2072	0.058*	
C49	0.83240 (17)	0.9449 (3)	0.2418 (7)	0.0632 (16)	
H49	0.8256	0.9552	0.1847	0.076*	
C50	0.81468 (18)	0.9721 (3)	0.3113 (6)	0.0602 (18)	
C51	0.8256 (2)	0.9552 (3)	0.3948 (5)	0.0631 (19)	
H51	0.8140	0.9730	0.4432	0.076*	
C52	0.8533 (2)	0.9124 (3)	0.4081 (4)	0.0557 (17)	
H52	0.8602	0.9016	0.4650	0.067*	
Gd1	0.914673 (6)	0.583155 (8)	0.24786 (4)	0.02827 (7)	
N1	1.02869 (15)	0.7404 (2)	0.6662 (3)	0.0519 (13)	
H1A	1.0461	0.7264	0.7017	0.062*	
H1B	1.0140	0.7687	0.6824	0.062*	
N2	0.7594 (2)	0.5425 (3)	0.0739 (6)	0.0738 (19)	
N3	0.9470 (2)	0.5368 (3)	0.5235 (4)	0.0637 (19)	
N4	0.9609 (2)	0.5518 (3)	0.9875 (4)	0.0626 (17)	
N5	0.88924 (17)	0.6679 (3)	0.9780 (4)	0.0525 (15)	
N6	0.87160 (17)	0.6623 (3)	0.5119 (4)	0.0558 (15)	
N7	0.78776 (18)	1.0158 (3)	0.2970 (5)	0.088 (2)	
H7A	0.7820	1.0262	0.2442	0.106*	
H7B	0.7767	1.0328	0.3409	0.106*	
N8	0.80819 (19)	0.6914 (3)	0.1722 (5)	0.0732 (17)	
N9	0.66714 (17)	0.9169 (2)	0.1790 (4)	0.0606 (14)	
N10	0.8535 (2)	0.9358 (3)	0.9186 (5)	0.0723 (17)	
N11	0.72363 (19)	0.6974 (3)	0.9154 (5)	0.0815 (19)	
O1	0.97175 (11)	0.64181 (16)	0.2889 (3)	0.0445 (9)	
O2	1.03318 (10)	0.68583 (14)	0.2462 (4)	0.0483 (8)	
O3	1.03391 (12)	0.59050 (16)	0.3044 (3)	0.0515 (10)	
O5	0.88950 (15)	0.77808 (18)	0.3359 (4)	0.0797 (16)	
O6	0.93894 (13)	0.84568 (19)	0.2913 (3)	0.0678 (13)	
O14	0.7721 (3)	0.5527 (4)	0.0030 (7)	0.185 (5)	
O15	0.7793 (2)	0.5406 (3)	0.1387 (7)	0.155 (4)	
O16	0.7249 (2)	0.5362 (4)	0.0840 (6)	0.143 (3)	
O20	0.92070 (18)	0.8376 (2)	0.4442 (3)	0.0874 (17)	
O1W	0.88775 (12)	0.65990 (17)	0.3333 (2)	0.0464 (10)	
O2W	0.86855 (12)	0.54534 (19)	0.3511 (3)	0.0533 (11)	
O3W	0.85363 (11)	0.59444 (18)	0.1683 (3)	0.0483 (10)	
O4W	0.89860 (12)	0.49138 (17)	0.1797 (3)	0.0485 (10)	
O5W	0.96918 (11)	0.54819 (17)	0.1606 (2)	0.0418 (9)	
O6W	0.95002 (14)	0.52250 (18)	0.3461 (2)	0.0468 (10)	
O7W	0.91889 (12)	0.66144 (19)	0.1469 (3)	0.0490 (11)	
O8W	0.7948 (2)	0.5832 (3)	0.3771 (5)	0.0923 (19)	
O9W	0.94198 (16)	0.8969 (2)	0.6030 (3)	0.0660 (13)	
S2	1.01437 (4)	0.64533 (6)	0.30545 (9)	0.0351 (3)	
S5	0.90793 (5)	0.83258 (7)	0.35428 (12)	0.0509 (4)	
H1W	0.8955 (12)	0.6538 (11)	0.3835 (6)	0.076*	
H2W	0.8968 (11)	0.68906 (11)	0.3108 (18)	0.076*	
H3W	0.8467 (2)	0.5584 (15)	0.3374 (17)	0.076*	
H6W	0.8437 (6)	0.6238 (9)	0.1882 (18)	0.076*	
H8W	0.8762 (5)	0.4857 (9)	0.199 (2)	0.076*	
H9W	0.9625 (6)	0.5555 (17)	0.1100 (2)	0.076*	
H10W	0.9884 (2)	0.5665 (14)	0.178 (2)	0.076*	
H11W	0.955 (2)	0.4895 (9)	0.331 (3)	0.076*	
H12W	0.950 (2)	0.527 (2)	0.3995 (6)	0.076*	
H14W	0.89570 (17)	0.6682 (13)	0.133 (2)	0.076*	
H16W	0.789 (2)	0.6161 (9)	0.390 (4)	0.076*	
H17W	0.937 (2)	0.884 (2)	0.5539 (16)	0.076*	
H18W	0.943 (2)	0.8730 (18)	0.642 (3)	0.076*	
H15W	0.793 (2)	0.5599 (18)	0.417 (3)	0.06 (3)*	
H5W	0.8410 (5)	0.5651 (7)	0.179 (2)	0.14 (4)*	
H4W	0.8703 (9)	0.51038 (19)	0.347 (2)	0.22 (7)*	
H7W	0.9160 (8)	0.4709 (4)	0.201 (2)	0.15 (5)*	
H13W	0.9299 (13)	0.6871 (7)	0.1746 (11)	0.15 (5)*	

### Atomic displacement parameters (Å^2^)

**Table:**

	*U*^11^	*U*^22^	*U*^33^	*U*^12^	*U*^13^	*U*^23^
C1	0.031 (3)	0.034 (3)	0.030 (3)	−0.004 (2)	−0.001 (2)	−0.001 (2)
C2	0.038 (3)	0.038 (3)	0.036 (3)	0.010 (2)	−0.001 (2)	0.000 (2)
C3	0.044 (3)	0.049 (4)	0.034 (3)	0.001 (3)	−0.014 (3)	0.003 (3)
C4	0.039 (3)	0.038 (3)	0.033 (3)	−0.006 (3)	0.003 (2)	−0.004 (2)
C5	0.040 (3)	0.036 (3)	0.041 (4)	0.006 (3)	−0.001 (3)	−0.009 (3)
C6	0.038 (3)	0.038 (3)	0.042 (3)	0.005 (2)	−0.005 (2)	0.000 (3)
C7	0.062 (5)	0.076 (6)	0.057 (5)	−0.003 (4)	0.009 (4)	0.022 (4)
C8	0.064 (5)	0.069 (5)	0.040 (4)	−0.021 (4)	−0.001 (4)	−0.001 (4)
C9	0.041 (4)	0.040 (4)	0.039 (3)	−0.002 (3)	0.009 (3)	0.005 (3)
C10	0.124 (7)	0.049 (5)	0.035 (4)	−0.016 (4)	0.002 (4)	0.003 (3)
C11	0.142 (8)	0.058 (5)	0.049 (5)	−0.013 (5)	−0.007 (5)	−0.005 (4)
C12	0.038 (3)	0.045 (4)	0.027 (3)	0.005 (3)	−0.001 (3)	0.001 (3)
C13	0.063 (5)	0.080 (6)	0.051 (4)	−0.030 (4)	0.003 (4)	−0.008 (4)
C14	0.072 (5)	0.108 (7)	0.050 (5)	−0.028 (5)	−0.012 (4)	−0.021 (5)
C15	0.110 (7)	0.078 (7)	0.034 (5)	−0.015 (5)	0.009 (4)	0.001 (4)
C16	0.075 (5)	0.072 (5)	0.041 (4)	−0.029 (4)	0.006 (4)	−0.006 (4)
C17	0.070 (5)	0.057 (5)	0.041 (4)	−0.012 (4)	0.001 (3)	0.017 (4)
C18	0.056 (4)	0.047 (4)	0.057 (5)	−0.019 (4)	−0.011 (4)	0.001 (4)
C19	0.041 (4)	0.038 (4)	0.029 (3)	−0.001 (3)	−0.008 (3)	0.006 (3)
C20	0.058 (4)	0.067 (5)	0.041 (4)	−0.024 (4)	0.000 (3)	0.010 (3)
C21	0.070 (5)	0.073 (5)	0.047 (4)	−0.027 (4)	−0.018 (4)	0.003 (4)
C22	0.036 (4)	0.040 (4)	0.042 (4)	0.011 (3)	−0.003 (3)	0.001 (4)
C23	0.080 (5)	0.060 (5)	0.028 (4)	−0.022 (4)	−0.002 (3)	−0.001 (3)
C24	0.081 (6)	0.071 (6)	0.051 (5)	−0.028 (5)	0.003 (4)	−0.009 (4)
C25	0.066 (5)	0.043 (4)	0.042 (4)	−0.010 (3)	0.000 (3)	0.004 (3)
C26	0.075 (5)	0.055 (5)	0.045 (4)	0.002 (4)	0.006 (4)	0.011 (4)
C27	0.044 (4)	0.108 (7)	0.078 (5)	−0.015 (5)	−0.005 (4)	0.012 (5)
C28	0.048 (4)	0.067 (5)	0.081 (5)	0.006 (4)	0.008 (4)	0.019 (4)
C29	0.051 (4)	0.048 (4)	0.057 (4)	0.003 (3)	0.004 (3)	0.004 (3)
C30	0.050 (4)	0.054 (4)	0.103 (6)	−0.007 (3)	−0.016 (4)	0.012 (4)
C31	0.067 (5)	0.057 (5)	0.123 (8)	−0.014 (4)	−0.013 (5)	0.013 (5)
C32	0.047 (4)	0.047 (4)	0.058 (4)	0.000 (3)	−0.001 (3)	0.018 (3)
C33	0.080 (5)	0.052 (4)	0.085 (6)	0.005 (4)	0.028 (4)	0.007 (4)
C34	0.106 (6)	0.047 (4)	0.081 (5)	−0.002 (4)	0.017 (5)	−0.003 (4)
C35	0.051 (4)	0.059 (5)	0.066 (5)	0.005 (4)	−0.004 (3)	0.013 (4)
C36	0.047 (4)	0.047 (4)	0.075 (5)	0.004 (3)	−0.012 (3)	−0.002 (4)
C37	0.045 (4)	0.060 (5)	0.109 (6)	0.004 (3)	0.004 (4)	−0.007 (4)
C38	0.068 (5)	0.055 (5)	0.119 (7)	0.010 (4)	−0.010 (5)	−0.010 (5)
C39	0.042 (4)	0.086 (6)	0.089 (6)	0.006 (4)	−0.010 (4)	−0.014 (5)
C40	0.046 (4)	0.049 (4)	0.090 (5)	0.006 (3)	−0.017 (4)	−0.001 (4)
C41	0.046 (4)	0.054 (4)	0.050 (4)	0.001 (3)	−0.007 (3)	−0.006 (3)
C42	0.039 (3)	0.053 (4)	0.041 (3)	0.002 (3)	−0.005 (3)	0.003 (3)
C43	0.044 (4)	0.065 (5)	0.061 (4)	0.007 (3)	−0.006 (3)	−0.008 (3)
C44	0.041 (4)	0.078 (6)	0.069 (5)	0.008 (4)	−0.008 (3)	0.008 (4)
C45	0.041 (3)	0.049 (4)	0.073 (4)	−0.005 (3)	−0.002 (3)	0.006 (3)
C46	0.058 (4)	0.045 (4)	0.084 (5)	0.005 (3)	0.001 (3)	0.001 (3)
C47	0.047 (3)	0.036 (3)	0.047 (4)	−0.008 (3)	0.006 (3)	0.000 (3)
C48	0.055 (3)	0.046 (3)	0.045 (3)	−0.005 (2)	−0.001 (4)	0.003 (4)
C49	0.060 (3)	0.057 (4)	0.073 (4)	−0.001 (3)	−0.016 (5)	−0.008 (5)
C50	0.040 (3)	0.041 (4)	0.099 (6)	−0.007 (3)	−0.002 (4)	−0.012 (4)
C51	0.061 (4)	0.058 (5)	0.070 (5)	−0.004 (4)	0.017 (4)	−0.009 (4)
C52	0.059 (4)	0.058 (4)	0.049 (4)	−0.006 (3)	0.012 (3)	0.007 (3)
Gd1	0.03323 (11)	0.02543 (11)	0.02617 (10)	−0.00137 (9)	0.00040 (18)	−0.00023 (17)
N1	0.067 (3)	0.053 (3)	0.036 (3)	0.003 (3)	−0.008 (2)	−0.011 (2)
N2	0.064 (4)	0.056 (4)	0.101 (6)	0.008 (3)	−0.026 (4)	−0.001 (4)
N3	0.093 (5)	0.069 (5)	0.028 (3)	0.011 (4)	0.002 (3)	0.005 (3)
N4	0.069 (4)	0.077 (5)	0.042 (3)	0.011 (3)	−0.006 (3)	−0.008 (3)
N5	0.056 (3)	0.058 (4)	0.043 (3)	−0.007 (3)	−0.009 (3)	0.011 (3)
N6	0.063 (4)	0.070 (4)	0.035 (3)	−0.005 (3)	0.004 (3)	−0.006 (3)
N7	0.078 (4)	0.063 (4)	0.124 (6)	0.023 (4)	−0.013 (4)	−0.020 (4)
N8	0.065 (4)	0.067 (4)	0.087 (5)	0.022 (3)	−0.015 (3)	−0.005 (4)
N9	0.059 (4)	0.056 (4)	0.067 (4)	0.010 (3)	0.003 (3)	0.008 (3)
N10	0.081 (4)	0.055 (4)	0.081 (4)	−0.014 (3)	0.009 (4)	0.010 (3)
N11	0.064 (4)	0.076 (5)	0.104 (5)	−0.019 (4)	−0.007 (4)	0.011 (4)
O1	0.042 (2)	0.044 (2)	0.047 (2)	−0.0058 (18)	−0.0118 (17)	−0.0100 (18)
O2	0.056 (2)	0.052 (2)	0.0373 (17)	−0.0096 (16)	0.003 (3)	0.003 (3)
O3	0.063 (3)	0.041 (2)	0.051 (2)	0.012 (2)	0.000 (2)	−0.0114 (19)
O5	0.081 (3)	0.033 (3)	0.125 (5)	−0.003 (2)	−0.007 (3)	−0.002 (3)
O6	0.054 (3)	0.068 (3)	0.082 (3)	0.004 (2)	0.013 (2)	−0.004 (3)
O14	0.277 (14)	0.140 (7)	0.136 (7)	0.073 (7)	0.105 (9)	0.027 (7)
O15	0.138 (6)	0.094 (5)	0.232 (10)	−0.034 (5)	−0.109 (7)	0.059 (6)
O16	0.073 (5)	0.174 (8)	0.181 (8)	−0.017 (5)	−0.017 (5)	−0.019 (7)
O20	0.109 (5)	0.089 (4)	0.064 (3)	0.024 (3)	−0.029 (3)	−0.009 (3)
O1W	0.069 (3)	0.039 (2)	0.031 (2)	−0.001 (2)	0.010 (2)	−0.0043 (17)
O2W	0.053 (3)	0.041 (3)	0.066 (3)	0.004 (2)	0.020 (2)	0.015 (2)
O3W	0.039 (2)	0.036 (2)	0.070 (3)	0.0021 (19)	−0.010 (2)	0.000 (2)
O4W	0.040 (2)	0.039 (2)	0.066 (3)	−0.002 (2)	0.005 (2)	−0.010 (2)
O5W	0.038 (2)	0.052 (3)	0.036 (2)	−0.0039 (19)	−0.0014 (17)	−0.0115 (19)
O6W	0.066 (3)	0.046 (3)	0.028 (2)	0.010 (2)	−0.002 (2)	0.0045 (18)
O7W	0.055 (3)	0.052 (3)	0.040 (2)	−0.012 (2)	−0.017 (2)	0.016 (2)
O8W	0.078 (4)	0.086 (5)	0.113 (5)	0.030 (4)	0.004 (4)	−0.014 (5)
O9W	0.078 (3)	0.069 (3)	0.052 (3)	0.009 (3)	0.004 (3)	0.004 (2)
S2	0.0392 (7)	0.0337 (7)	0.0324 (7)	−0.0002 (6)	−0.0037 (6)	−0.0052 (6)
S5	0.0543 (9)	0.0388 (9)	0.0594 (10)	−0.0008 (7)	−0.0002 (8)	−0.0030 (7)

### Geometric parameters (Å, °)

**Table:**

C1—C2	1.371 (7)	C35—H35	0.9300
C1—C6	1.389 (7)	C36—H36	0.9300
C1—S2	1.763 (5)	C37—C38	1.367 (9)
C2—C3	1.380 (8)	C37—C41	1.384 (9)
C2—H2	0.9300	C37—H37	0.9300
C3—C4	1.396 (8)	C38—N8	1.314 (9)
C3—H3	0.9300	C38—H38	0.9300
C4—N1	1.382 (7)	C39—N8	1.335 (9)
C4—C5	1.388 (8)	C39—C40	1.361 (9)
C5—C6	1.372 (8)	C39—H39	0.9300
C5—H5	0.9300	C40—C41	1.382 (9)
C6—H6	0.9300	C40—H40	0.9300
C7—N3	1.324 (10)	C41—C42	1.468 (8)
C7—C8	1.380 (11)	C42—C43	1.396 (8)
C7—H7	0.9300	C42—C45	1.391 (8)
C8—C9	1.380 (9)	C43—C44	1.369 (9)
C8—H8	0.9300	C43—H43	0.9300
C9—C10	1.380 (10)	C44—N9	1.317 (9)
C9—C12	1.465 (8)	C44—H44	0.9300
C10—C11	1.379 (10)	C45—C46	1.389 (9)
C10—H10	0.9300	C45—H45	0.9300
C11—N3	1.312 (10)	C46—N9	1.316 (9)
C11—H11	0.9300	C46—H46	0.9300
C12—C13	1.361 (9)	C47—C48	1.355 (10)
C12—C16	1.373 (9)	C47—C52	1.372 (8)
C13—C14	1.372 (11)	C47—S5	1.773 (6)
C13—H13	0.9300	C48—C49	1.382 (8)
C14—N4	1.302 (10)	C48—H48	0.9300
C14—H14	0.9300	C49—C50	1.369 (11)
C15—N4	1.302 (10)	C49—H49	0.9300
C15—C16	1.391 (10)	C50—N7	1.381 (8)
C15—H15	0.9300	C50—C51	1.378 (10)
C16—H16	0.9300	C51—C52	1.381 (10)
C17—N5	1.325 (9)	C51—H51	0.9300
C17—C18	1.376 (10)	C52—H52	0.9300
C17—H17	0.9300	Gd1—O6W	2.375 (4)
C18—C19	1.377 (9)	Gd1—O2W	2.373 (4)
C18—H18	0.9300	Gd1—O1W	2.389 (4)
C19—C20	1.392 (9)	Gd1—O3W	2.392 (4)
C19—C22	1.489 (6)	Gd1—O7W	2.391 (4)
C20—C21	1.383 (10)	Gd1—O5W	2.401 (4)
C20—H20	0.9300	Gd1—O1	2.434 (4)
C21—N5	1.307 (9)	Gd1—O4W	2.440 (4)
C21—H21	0.9300	N1—H1A	0.8600
C22—C25	1.388 (9)	N1—H1B	0.8600
C22—C23	1.377 (9)	N2—O16	1.176 (8)
C23—C24	1.384 (10)	N2—O14	1.184 (10)
C23—H23	0.9300	N2—O15	1.190 (9)
C24—N6	1.322 (10)	N7—H7A	0.8600
C24—H24	0.9300	N7—H7B	0.8600
C25—C26	1.376 (9)	O1—S2	1.453 (4)
C25—H25	0.9300	O2—S2	1.450 (4)
C26—N6	1.320 (9)	O3—S2	1.438 (4)
C26—H26	0.9300	O5—S5	1.441 (5)
C27—N11	1.322 (10)	O6—S5	1.446 (5)
C27—C28	1.381 (10)	O20—S5	1.437 (5)
C27—H27	0.9300	O1W—H1W	0.819 (17)
C28—C29	1.375 (9)	O1W—H2W	0.820 (19)
C28—H28	0.9300	O2W—H3W	0.820 (17)
C29—C30	1.376 (9)	O2W—H4W	0.820 (7)
C29—C32	1.481 (9)	O3W—H6W	0.82 (2)
C30—C31	1.380 (10)	O3W—H5W	0.821 (18)
C30—H30	0.9300	O4W—H8W	0.82 (2)
C31—N11	1.321 (9)	O4W—H7W	0.82 (2)
C31—H31	0.9300	O5W—H9W	0.819 (11)
C32—C36	1.385 (9)	O5W—H10W	0.82 (2)
C32—C33	1.377 (10)	O6W—H11W	0.82 (3)
C33—C34	1.395 (10)	O6W—H12W	0.819 (12)
C33—H33	0.9300	O8W—H16W	0.82 (3)
C34—N10	1.310 (10)	O8W—H15W	0.82 (4)
C34—H34	0.9300	O9W—H17W	0.82 (3)
C35—N10	1.335 (9)	O9W—H18W	0.82 (4)
C35—C36	1.369 (9)		
			
C2—C1—C6	119.1 (5)	C39—C40—C41	120.9 (7)
C2—C1—S2	120.7 (4)	C39—C40—H40	119.5
C6—C1—S2	120.2 (4)	C41—C40—H40	119.5
C1—C2—C3	120.6 (5)	C37—C41—C40	114.7 (6)
C1—C2—H2	119.7	C37—C41—C42	122.7 (6)
C3—C2—H2	119.7	C40—C41—C42	122.6 (6)
C2—C3—C4	120.7 (5)	C43—C42—C45	116.4 (6)
C2—C3—H3	119.6	C43—C42—C41	121.5 (6)
C4—C3—H3	119.6	C45—C42—C41	122.1 (5)
N1—C4—C3	120.4 (5)	C44—C43—C42	118.8 (7)
N1—C4—C5	121.5 (5)	C44—C43—H43	120.6
C3—C4—C5	118.1 (5)	C42—C43—H43	120.6
C6—C5—C4	120.9 (5)	N9—C44—C43	125.0 (7)
C6—C5—H5	119.5	N9—C44—H44	117.5
C4—C5—H5	119.5	C43—C44—H44	117.5
C5—C6—C1	120.5 (5)	C46—C45—C42	119.6 (6)
C5—C6—H6	119.7	C46—C45—H45	120.2
C1—C6—H6	119.7	C42—C45—H45	120.2
N3—C7—C8	124.3 (7)	N9—C46—C45	123.3 (7)
N3—C7—H7	117.9	N9—C46—H46	118.3
C8—C7—H7	117.9	C45—C46—H46	118.3
C7—C8—C9	120.4 (7)	C48—C47—C52	118.6 (6)
C7—C8—H8	119.8	C48—C47—S5	120.9 (5)
C9—C8—H8	119.8	C52—C47—S5	120.4 (5)
C8—C9—C10	115.0 (6)	C47—C48—C49	121.3 (8)
C8—C9—C12	122.8 (7)	C47—C48—H48	119.3
C10—C9—C12	122.2 (7)	C49—C48—H48	119.3
C11—C10—C9	120.5 (7)	C50—C49—C48	120.9 (9)
C11—C10—H10	119.8	C50—C49—H49	119.6
C9—C10—H10	119.8	C48—C49—H49	119.6
N3—C11—C10	124.4 (8)	C49—C50—N7	120.4 (8)
N3—C11—H11	117.8	C49—C50—C51	117.6 (7)
C10—C11—H11	117.8	N7—C50—C51	122.0 (7)
C13—C12—C16	116.4 (6)	C52—C51—C50	121.3 (7)
C13—C12—C9	122.8 (7)	C52—C51—H51	119.3
C16—C12—C9	120.7 (7)	C50—C51—H51	119.3
C12—C13—C14	120.0 (7)	C51—C52—C47	120.2 (6)
C12—C13—H13	120.0	C51—C52—H52	119.9
C14—C13—H13	120.0	C47—C52—H52	119.9
N4—C14—C13	124.5 (7)	O6W—Gd1—O2W	71.80 (15)
N4—C14—H14	117.8	O6W—Gd1—O1W	107.07 (14)
C13—C14—H14	117.8	O2W—Gd1—O1W	70.93 (15)
N4—C15—C16	124.5 (8)	O6W—Gd1—O3W	144.17 (16)
N4—C15—H15	117.7	O2W—Gd1—O3W	79.51 (15)
C16—C15—H15	117.7	O1W—Gd1—O3W	82.48 (14)
C12—C16—C15	118.6 (7)	O6W—Gd1—O7W	146.10 (15)
C12—C16—H16	120.7	O2W—Gd1—O7W	138.36 (14)
C15—C16—H16	120.7	O1W—Gd1—O7W	78.38 (14)
N5—C17—C18	123.2 (7)	O3W—Gd1—O7W	68.99 (14)
N5—C17—H17	118.4	O6W—Gd1—O5W	76.42 (13)
C18—C17—H17	118.4	O2W—Gd1—O5W	137.30 (14)
C17—C18—C19	121.5 (7)	O1W—Gd1—O5W	147.41 (14)
C17—C18—H18	119.2	O3W—Gd1—O5W	114.14 (14)
C19—C18—H18	119.2	O7W—Gd1—O5W	81.94 (14)
C20—C19—C18	115.0 (6)	O6W—Gd1—O1	77.38 (15)
C20—C19—C22	121.0 (7)	O2W—Gd1—O1	123.49 (14)
C18—C19—C22	124.0 (7)	O1W—Gd1—O1	74.70 (13)
C21—C20—C19	118.9 (7)	O3W—Gd1—O1	137.76 (14)
C21—C20—H20	120.5	O7W—Gd1—O1	71.85 (14)
C19—C20—H20	120.5	O5W—Gd1—O1	74.60 (13)
N5—C21—C20	125.7 (7)	O6W—Gd1—O4W	81.63 (15)
N5—C21—H21	117.2	O2W—Gd1—O4W	79.08 (15)
C20—C21—H21	117.1	O1W—Gd1—O4W	143.66 (14)
C25—C22—C23	116.3 (7)	O3W—Gd1—O4W	72.10 (15)
C25—C22—C19	122.6 (7)	O7W—Gd1—O4W	114.26 (15)
C23—C22—C19	121.1 (7)	O5W—Gd1—O4W	68.59 (13)
C24—C23—C22	119.1 (7)	O1—Gd1—O4W	140.92 (13)
C24—C23—H23	120.4	C4—N1—H1A	120.0
C22—C23—H23	120.4	C4—N1—H1B	120.0
N6—C24—C23	124.4 (8)	H1A—N1—H1B	120.0
N6—C24—H24	117.8	O16—N2—O14	119.8 (10)
C23—C24—H24	117.8	O16—N2—O15	116.1 (10)
C22—C25—C26	120.2 (7)	O14—N2—O15	124.0 (10)
C22—C25—H25	119.9	C7—N3—C11	115.4 (7)
C26—C25—H25	119.9	C14—N4—C15	115.8 (7)
N6—C26—C25	123.4 (7)	C21—N5—C17	115.6 (6)
N6—C26—H26	118.3	C26—N6—C24	116.5 (6)
C25—C26—H26	118.3	C50—N7—H7A	120.0
N11—C27—C28	124.1 (7)	C50—N7—H7B	120.0
N11—C27—H27	118.0	H7A—N7—H7B	120.0
C28—C27—H27	118.0	C38—N8—C39	116.3 (6)
C29—C28—C27	119.2 (7)	C38—N8—H14W	158.5 (9)
C29—C28—H28	120.4	C39—N8—H14W	83.6 (7)
C27—C28—H28	120.4	C46—N9—C44	116.9 (6)
C30—C29—C28	117.1 (6)	C34—N10—C35	116.3 (7)
C30—C29—C32	120.5 (6)	C27—N11—C31	116.1 (7)
C28—C29—C32	122.4 (6)	S2—O1—Gd1	148.1 (2)
C31—C30—C29	119.3 (7)	S2—O2—H13W	77.1 (2)
C31—C30—H30	120.4	Gd1—O1W—H1W	105 (2)
C29—C30—H30	120.4	Gd1—O1W—H2W	104.8 (18)
N11—C31—C30	124.1 (8)	H1W—O1W—H2W	115 (3)
N11—C31—H31	118.0	Gd1—O2W—H3W	106.0 (19)
C30—C31—H31	118.0	Gd1—O2W—H4W	106 (2)
C36—C32—C33	117.4 (6)	H3W—O2W—H4W	115 (3)
C36—C32—C29	119.6 (6)	Gd1—O3W—H6W	104.7 (17)
C33—C32—C29	123.0 (6)	Gd1—O3W—H5W	104.4 (16)
C32—C33—C34	118.5 (7)	H6W—O3W—H5W	115 (2)
C32—C33—H33	120.7	Gd1—O4W—H8W	101.1 (17)
C34—C33—H33	120.7	Gd1—O4W—H7W	100.8 (14)
N10—C34—C33	124.4 (8)	H8W—O4W—H7W	115 (3)
N10—C34—H34	117.8	Gd1—O5W—H9W	103.9 (17)
C33—C34—H34	117.8	Gd1—O5W—H10W	104.1 (18)
N10—C35—C36	124.0 (7)	H9W—O5W—H10W	114 (3)
N10—C35—H35	118.0	Gd1—O6W—H11W	118 (4)
C36—C35—H35	118.0	Gd1—O6W—H12W	122 (4)
C35—C36—C32	119.4 (7)	H11W—O6W—H12W	114 (5)
C35—C36—H36	120.3	H16W—O8W—H15W	116 (5)
C32—C36—H36	120.3	H17W—O9W—H18W	115 (4)
C38—C37—C41	121.1 (7)	O3—S2—O2	112.1 (2)
C38—C37—H37	119.4	O3—S2—O1	113.3 (2)
C41—C37—H37	119.4	O2—S2—O1	110.9 (2)
N8—C38—C37	123.5 (7)	O3—S2—C1	107.7 (2)
N8—C38—H38	118.3	O2—S2—C1	107.1 (3)
C37—C38—H38	118.3	O1—S2—C1	105.3 (2)
N8—C39—C40	123.5 (7)	O20—S5—O6	113.5 (3)
N8—C39—H39	118.3	O20—S5—O5	112.6 (4)
C40—C39—H39	118.3	O6—S5—O5	111.5 (3)
N8—C39—H14W	71.8 (5)	O20—S5—C47	106.7 (3)
C40—C39—H14W	162.6 (6)	O6—S5—C47	105.2 (3)
H39—C39—H14W	47.3	O5—S5—C47	106.7 (3)

### Hydrogen-bond geometry (Å, °)

**Table:**

*D*—H···*A*	*D*—H	H···*A*	*D*···*A*	*D*—H···*A*
O1W—H1W···N6	0.82 (2)	2.12 (2)	2.770 (7)	136 (3)
O1W—H2W···O5	0.82 (2)	2.13 (1)	2.759 (6)	134 (3)
O2W—H3W···O8W	0.82 (2)	1.93 (1)	2.657 (7)	147 (2)
O3W—H6W···N8	0.82 (2)	1.99 (1)	2.728 (7)	149 (2)
O4W—H8W···N9^i^	0.82 (2)	2.19 (2)	2.807 (7)	133 (1)
O5W—H9W···N4^ii^	0.82 (1)	1.86 (1)	2.647 (7)	159 (2)
O5W—H10W···O3	0.82 (2)	2.51 (2)	3.236 (6)	148 (4)
O5W—H10W···O1	0.82 (2)	2.50 (3)	2.931 (5)	114 (2)
O6W—H11W···O3^iii^	0.82 (3)	1.95 (3)	2.765 (6)	175 (5)
O6W—H12W···N3	0.82 (1)	1.90 (1)	2.719 (7)	178 (8)
O7W—H13W···N1^iv^	0.82 (3)	2.19 (2)	2.902 (7)	145 (3)
O7W—H14W···N5^ii^	0.82 (1)	2.37 (4)	2.758 (7)	110 (3)
O7W—H14W···O3W	0.82 (1)	2.29 (1)	2.709 (6)	112 (3)
O8W—H16W···N11^v^	0.82 (3)	1.98 (3)	2.798 (9)	176 (6)
O9W—H17W···O20	0.82 (3)	2.06 (4)	2.873 (7)	169 (6)
O9W—H18W···O2^vi^	0.82 (4)	2.24 (5)	3.028 (7)	161 (7)
N1—H1A···O6^vi^	0.86	2.22	2.972 (7)	146
N1—H1B···O2^vi^	0.86	2.14	2.958 (6)	159
N7—H7B···O14^vii^	0.86	2.51	3.289 (12)	151
N7—H7A···O15^viii^	0.86	2.63	3.345 (12)	141
N7—H7A···O16^viii^	0.86	2.46	3.302 (13)	167

Symmetry codes: (i) −*x*+3/2, *y*−1/2, *z*; (ii) *x*, *y*, *z*−1; (iii) −*x*+2, −*y*+1, *z*; (iv) −*x*+2, −*y*+3/2, *z*−1/2; (v) −*x*+3/2, *y*, *z*−1/2; (vi) −*x*+2, −*y*+3/2, *z*+1/2; (vii) *x*, *y*+1/2, *z*+1/2; (viii) −*x*+3/2, *y*+1/2, *z*.
